# Effect of Different Dosages of ST36 Indirect Moxibustion on the Skin Temperature of the Lower Legs and Feet

**DOI:** 10.3390/medicines5020057

**Published:** 2018-06-15

**Authors:** Hiroshi Kuge, Hidetoshi Mori, Tateyuki Morisawa, Kazuyo Hanyu, Junji Miyazaki, Mayumi Watanabe, Tim Hideaki Tanaka

**Affiliations:** 1 Course of Acupuncture and Moxibustion, Department of Health, Faculty of Health Science, Tsukuba University of Technology, Tsukuba 305-0821, Japan; sherry@kha.biglobe.ne.jp (H.K.); hmori@k.tsukuba-tech.ac.jp (H.M.); morisawat@ipu.ac.jp (T.M.); drh2014@yahoo.co.jp (K.H.); torajiro@ruby.famille.ne.jp (M.W.); 2 Anesthesiology Department of Osaka Medical College Hospital, Osaka 569-8686, Japan; 3 Center for Humanities and Sciences, Ibaraki Prefectural University of Health Sciences, Ibaraki 300-0394, Japan; 4 Department of Environmental and Natural Resource Sciences, Tokyo University of Agriculture and Technology, Laboratory of Plant Morphology and Bio-Material Physics, Researcher of Collaborative Industry-Alliance-Government, Tokyo 183-8509, Japan; 5 Public Health, Department of Social Medicine, Graduate School of Medicine, Osaka University, Osaka 565-0871, Japan; j_miyazaki@kumamoto-u.ac.jp

**Keywords:** moxibustion, thermography, skin temperature, acupuncture points

## Abstract

**Background:** Indirect moxibustion (IM) has been previously performed between the spinous process while recording skin temperature of the trunk. However, moxibustion is often applied not only to acupuncture points on the back, but also to points located on the limbs. Thus, there is a need to investigate skin temperature (ST) responses following IM applied to the limbs. **Methods:** In Experiment 1 (Exp 1), subjects were randomly assigned to three groups: the left IM stimulation, right IM stimulation and control groups. In Experiment 2 (Exp 2), the subjects underwent two experimental sessions consisting of a single stimulation of IM or triple stimulations of IM. The IM stimulation was administered to the ST36 acupuncture point. A thermograph was used to obtain the ST on the lower limbs. **Results:** In Exp 1, the ST of the lower limbs increased in the stimulation groups whereas there was no increase in the non-stimulation group. In Exp 2, no significant response occurred between the single and triple stimulation of IM groups for all observed sites except for the left ankle ST. **Conclusions:** Lower limb ST increased following IM application to the ST36 point. No difference was observed between the dosage of the stimulation and ST responses.

## 1. Introduction

Moxibustion is a form of thermal therapy that involves burning the herb *moxa* (*Artemisia princeps*, commonly called Japanese mugwort) over acupuncture points. One of the most commonly used moxibustion procedures is an indirect moxibustion (IM) method. The main advantage of IM is minimization of the possibility of a burn incident because the burning moxa does not directly come in contact with the skin [1,2]. There are different types of IM available. One example is a cylinder type, which contains dried moxa leaves inside a small cylindrical tube. 

In a previous study using the cylinder IM method on mice, the temperature reached 65 °C on the skin and 45 °C in the subcutaneous tissue [3]. In the human study, the skin temperature ranged from 46 °C to 67.5 °C following the application of cylinder IM [1]. The increased skin temperature, as seen in the previous studies, was thought to be induced by the excitation of skin nociceptors influencing peripheral blood vessels, thereby raising the skin temperature and blood flow in the skin tissue [4]. In another clinical study, IM applied to the lower leg and lumbar regions showed improvement in the quality of life of elderly patients [5].

We previously performed IM stimulation between the vertebrae of the spinous process and observed the skin temperature of the trunk [2] The results showed that there was no difference in the change in skin temperature between a single application and repeated applications of IM. It should be noted however that moxibustion is often applied not only to acupuncture points on the back, but also to points located on the upper and lower limbs. Thus, there is a need to investigate skin temperature responses following IM stimulation applied to the limbs.

In the present study, we conducted two experiments involving IM administration to the lower limbs. First, we conducted a preliminary experiment investigating the skin temperature responses from IM applications to the lower limbs with a no-stimulation control group (Experiment 1). Then, we conducted an experiment to investigate possible differences in skin temperature responses following a single or triple IM stimulation (Experiment 2). 

## 2. Materials and Methods

### 2.1. Participants

The participants were 48 healthy students (34 for Experiment 1 and 14 for Experiment 2). Their mean age was 22.36 ± 4.2 years. All participants were informed of the purpose and nature of the experiments, and a written consent was obtained from each participant, in compliance with the World Medical Association Declaration of Helsinki. The study was approved by the Research Ethics Committee of the Tsukuba University of Technology (Approval No. 241121, Approval date: 21 November 2012). 

### 2.2. Experiment 1

#### 2.2.1. Subjects

Thirty-four students (mean age 22.4 ± 4.4 years) were randomly assigned to three groups, the left IM stimulation group (*n* = 11), right IM stimulation group (*n* = 11) and control group (*n* = 12). 

#### 2.2.2. Stimulation Method

In both Experiments 1 and 2, the stimulation site was Stomach (ST) 36 (ST36) (acupuncture point located on the anterior aspect of the lower leg, about one finger-breadth lateral to the tibia, just inferior to the tibial tuberosity). Thermal stimulation was applied by using cylinder type IM (Kamaya Mini^®^, Kamaya Moxa Co., Ltd., Tokyo, Japan). Kamaya Mini^®^ consists of a cylindrical paper pipe (9 mm in diameter and 12 mm in height) filled with dried moxa. There is an 8-mm cavity between the moxa and the skin’s surface, permitting the indirect administration of thermal stimulation. A standardized amount of moxa is inserted in each Kamaya-mini so that the thermal intensity is a standardized amount for each piece. The heat emitted by a single application of Kamaya Mini^®^ lasted approximately three minutes. The subjects in the control group proceeded with the exact same sequences as the stimulation groups; however, the moxa was not ignited during the procedure.

#### 2.2.3. Measurement Procedure

A thermograph (JTG-5310, a quantum well infrared detector, wavelength: 8–13 μm, image resolution: 512 dots × 480 lines, temperature resolution: 0.05 °C, JEOL Ltd., Tokyo, Japan) was used to obtain the skin temperature on the legs and feet of the participants. Subjects were asked to lie quietly on the table in a supine position for 20 min. Thermographic images were then obtained at the following time periods: before (Pre) and immediately after the administration of IM (Post 0), as well as 5 (Post 5), 10 (Post 10), 15 (Post 15), and 20 (Post 20) minutes afterward.

**Figure 1:**
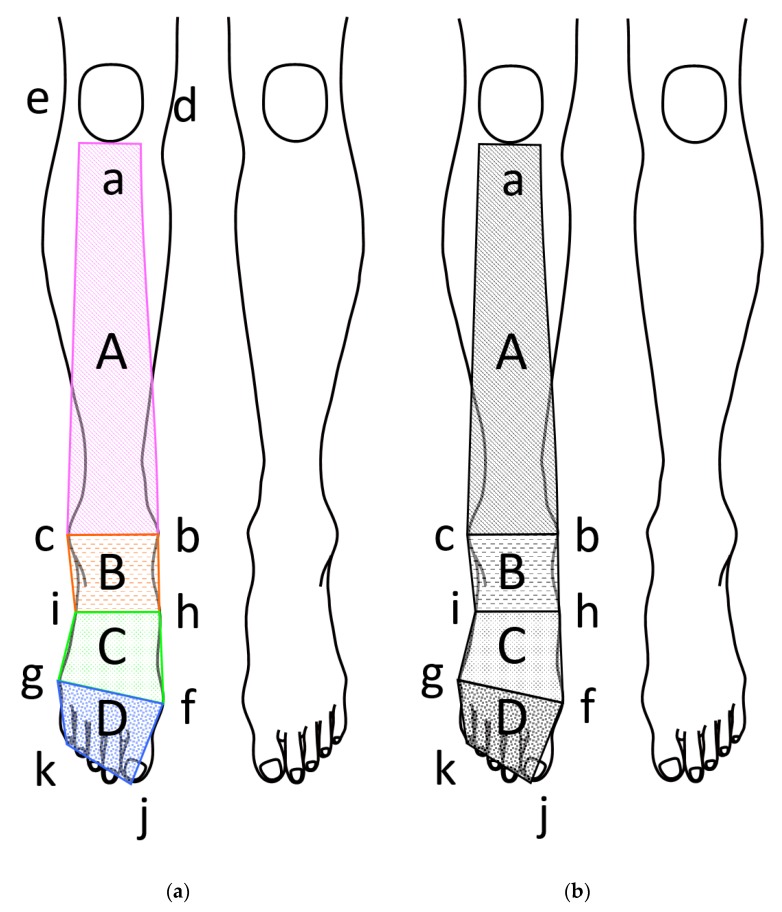
Arbitrary frames used to analyze skin temperature of lower limb. A. Anterior aspect of lower leg frame (referred as Lower Leg in the text). B. Anterior aspect of ankle frame (referred as Ankle in the text). C. Dorsal proximal aspect of foot frame (referred as Proximal Foot in the text). D. Dorsal distal aspect of foot frame (referred as Distal Foot in the text). Anatomical land marks used to create arbitrary frames. a. Inferior border of the patella. b. Highest point of the medial malleolus. c. Highest point of the lateral malleolus. d. Medial border of the patella. e. Lateral border of the patella. f. Medial aspect of the 1st metatarsophalangeal joint. g. Lateral aspect of the 5th metatarsophalangeal joint. h. Midpoint between b and f. i. Midpoint between c and g. j. Distal end of the 1st phalanx. k. Distal end of the 5th phalanx.

To analyze the skin temperature, four arbitrary frames were selected for each lower limb: the anterior aspect of the lower leg (hereafter referred to as *lower leg*), the anterior aspect of the ankle (hereafter referred to as *ankle*), the dorsal proximal aspect of the foot (hereafter referred to as *proximal foot*), and the dorsal distal aspect of the foot (hereafter referred to as *distal foot*). The exact locations of the frames, with anatomical landmarks, are shown in Figure 1. ST was recorded at the rate of 480 samples per second for 0.8 second. The samples were averaged and used for later analysis.

All experimental sessions were conducted under ambient room conditions of 26.1 ± 0.1 °C and 54.6 ± 0.2% humidity.

### 2.3. Experiment 2

#### 2.3.1. Subjects

Fourteen students (mean age 22.2 ± 1.1 years) were randomly divided into two groups by the envelope allocation method. One group (Group A, *n* = 7) received single stimulation of IM in the first experiment session and then received triple stimulations of IM in the second session. Another group (Group B, *n* = 7) completed the sessions in a reversed sequence. There was a one-week interval between the first and second experimental sessions.

#### 2.3.2. Stimulation Method

As indicated, the heat emitted by a single application of Kamaya Mini^®^ IM lasted approximately three minutes. Two different IM dosages were used for comparison: three minutes of stimulation (IM applied once—hereafter referred to as “single stimulation of IM”) and nine minutes of stimulation (IM applied three times—hereafter referred to as “triple stimulation of IM”). For triple stimulation of IM, the burned moxa was immediately replaced by a new one, thus there was virtually no time interval between the applications of moxa. The IM stimulation was administered on ST36 on the right side.

#### 2.3.3. Measurement Procedure

The skin temperature response was recorded in the same manner as in Experiment 1. All experimental sessions were conducted under ambient room conditions of 25.9 ± 0.1 °C and 54.6 ± 0.1% humidity.

### 2.4. Statistical Analysis

For Experiment 1, serial changes in skin temperature between the stimulation groups (left and right) and control group were analyzed via mixed-model three-way factorial ANOVA (left IM stimulation, right IM stimulation and control as the factors with six time levels). 

For Experiment 2, serial changes in skin temperature Group A and B were analyzed via mixed-model two-way factorial ANOVA (two sequence factors and six time levels). Serial changes in skin temperature between the stimulation groups were analyzed via mixed-model two-way factorial ANOVA (single stimulation of IM and triple stimulations of IM as the factors with six time levels). For both experiments, serial changes in skin temperature between the stimulated and the non-stimulated side were analyzed via mixed-model two-way factorial ANOVA (skin temperature on the stimulated and the non-stimulated side as the factors with six time levels). Serial changes in skin temperature within the group were analyzed via linear analysis using Bonferroni’s multiple-comparison tests. The significance threshold was set at 0.05. 

## 3. Results

### 3.1. Experiment 1

For skin temperature changes in the left and right lower legs, ankles, proximal feet, and distal feet regions, no significant interactions occurred between the left IM, right IM, and control groups (φ = 10, f = 0.265–0.541, *p* > 0.85). For skin temperature changes in the left IM and right IM, no significant response occurred between ipsilateral and contralateral sides (φ = 5, f = 0.024–0.189, *p* > 0.92). Skin temperature responses are summarized in Table 1.

#### 3.1.1. Left IM Stimulation Group

The skin temperature of the stimulated side of the leg increased after the IM stimulation in all measurement sites. Post stimulation skin temperature elevation was also observed in the non-stimulated side of the leg.

#### 3.1.2. Right IM Stimulation Group

The skin temperature of the stimulated side of the leg increased after the IM stimulation in all measurement sites. Post stimulation skin temperature elevation was also observed in the non-stimulated side of the leg.

#### 3.1.3. Control Group

No significant change was observed in the skin temperature in the control group.

### 3.2. Experiment 2

For serial changes in skin temperature between group A and B, no interaction was detected between the groups (φ = 5, f = 0.068–2.355, *p* > 0.05). For skin-temperature changes in the left and right lower legs, proximal feet, distal feet, and right ankle regions, no significant interactions occurred between the single stimulation of IM and triple stimulation of IM groups (φ = 5, f = 0.743–1.706, *p* > 0.010). There was an interaction between interventions and the left ankle (φ = 5, f = 2.551, *p* = 0.031). There was no significant difference between the interventions (φ = 1, f = 0.015, *p* = 0.903). There was a significant change between the measurement times (φ = 5, f = 28.273, *p* < 0.001).

Table 2 shows the skin temperature changes of the leg and foot following a single stimulation of IM and triple stimulation of IM. 

#### 3.2.1. Single Stimulation of IM

The skin temperature of the stimulated side of the leg increased after the single stimulation of IM in all measurement sites. Post stimulation skin temperature elevation was also observed in the non-stimulated side of the leg.

#### 3.2.2. Triple Stimulation of IM

The skin temperature of the stimulated side of the leg increased after the triple stimulation of IM in all measurement sites. Post stimulation skin temperature elevation was also observed in the non-stimulated side of the leg.

## 4. Discussion

Previously, we reported that no differences were manifested in the temperature curves and maximum temperatures with the use of single and triple applications of IM [1]. Further, the administration of a single IM dose on the three acupuncture points (Governing Vessel (GV)14, GV9, and GV4) produced greater skin temperature changes compared to the triple application of IM to the single point (GV14) only [2]. 

In contrast to our previous study, in which IM was applied on the posterior trunk surface, the stimulation was applied to the lower leg (ST36 point) in the present study. The ST36 point is one of the most commonly used acupuncture points in clinical and research settings [6,7,8]. Previous studies suggested that IM applied to the ST36 point influences various physiological functions such as the alteration of natural killer cells [9] and gastrointestinal motility [10].

In the present study, we first conducted a preliminary experiment to investigate the skin temperature responses between the IM applications to the left and right side of ST 36 and the control group (Experiment 1). It showed that skin temperature of the lower limbs increased in stimulation groups whereas no increase was observed in the non-stimulation group. It also confirmed that there was no difference in the skin temperature response between IM applications to the left and right sides of the limbs. It should be noted that the elevation of the skin temperature was observed on both the stimulated and non-stimulated sides. This bilateral response from the unilateral stimulation suggests that it might be inadequate to use the contralateral side of the stimulation as a control when conducting a moxibustion study.

In Experiment 2, we investigated the possible difference in temperature response between the single stimulation of IM and triple stimulation of IM groups. No significant response occurred between the two stimulation groups in all observed sites, except for the left ankle skin temperature. 

As seen in Experiment 1, the increase of skin temperature was observed on both the stimulated and non-stimulated sides. This phenomenon indicates that unilateral moxibustion stimulation induced both regional and systemic response likely via the axon and the supraspinal reflex. The potential involvement of the supraspinal reflex by thermal stimulation was also suggested in our previous experiment, in which an increase in skin temperature on the lower limbs was observed following an abdominal hot-stone administration [11]. A study by Su et al. [10]. demonstrated that thermal stimulation applied to the ST 36 point modulates gastric motility. They hypothesized that the response was induced via Aδ- and C-afferent fibers. Kawakita et al. [12] indicated that the axon reflex induced by somatosensory stimulation such as acupuncture or moxibustion is related to the activation of the polymodal receptor. We consider that the regional increase in skin temperature via IM observed in the present study also involved the axon reflex.

In previous clinical studies of moxibustion, some researchers applied IM with a handheld moxa-stick. In the series of clinical studies by Chen et al., they utilized a handheld moxa-stick IM method, referred to as heat-sensitive moxibustion [13,14,15,16], in which the intensity and dosage of thermal stimulation was adjusted on an individual basis according to each subject’s thermosensitization. In a study conducted on people with knee osteoarthritis for instance, a significant therapeutic improvement was observed among subjects who received heat-sensitive moxibustion compared to subjects who received the standard 15 min of IM [15]. The duration of heat-sensitive moxibustion ranged from 28 to 65 min with varied intensity. In this study, we used Kamaya Mini^®^ as the method of IM. It produced a fixed thermal intensity among all subjects. An important finding in this study was that varied thermal intensity could become a confounding factor when examining a repetitive dosage of IM. Nonetheless, both repetition of IM and intensity of emitted heat should be considered in clinical applications of moxibustion. Although serious complications from moxibustion are rare, burn incidents have been reported due to both direct and indirect forms of moxibustion [17]. Based on this study, we consider that it might be more clinically desirable, in terms of efficacy, safety, and efficiency, that each acupuncture point is thermally stimulated a single time, rather than applying stimulation to the same point multiple times.

There are some limitations to this study. In this study, skin temperature changes were only evaluated on young healthy participants. These results may not be applicable to other populations; for instance, among patients with Raynaud’s disease who have compromised peripheral blood flow. There was a significant interaction detected between interventions and the left ankle skin temperature. This response was considered an outlier. Whether it was due to random variation or other unknown events could not be determined. 

## 5. Conclusions

Lower limb skin temperature was elevated following IM application to the ST36 point. As no significant difference in ST response was observed between the dosages of the stimulation, the single stimulation of IM should be sufficient to elevate the ST. 

## Figures and Tables

**Table 1:** Skin temperature responses before and after indirect moxibustion (IM) to the left leg (LIM), right leg (RIM), and control group (CM).

Measurement Sites	Interventions	Pre	Post 0	Post 5	Post 10	Post 15	Post 20
Left Low Lg	(LIM)	33.0 ± 0.6	33.1 ± 0.6	33.1 ± 0.5	33.2 ± 0.5	33.3 ± 0.5 **	33.3 ± 0.5 **
	(RIM)	33.0 ± 0.7	32.9 ± 0.7	33.2 ± 0.7	33.3 ± 0.7	33.3 ± 0.8 *	33.3 ± 0.8 **
	(CM)	33.1 ± 0.7	33.1 ± 0.7	33.1 ± 0.6	33.1 ± 0.6	33.1 ± 0.6	33.1 ± 0.6
Right Low Lg	(LIM)	33.0 ± 0.6	33.0 ± 0.6	33.1 ± 0.5	33.2 ± 0.5 *	33.3 ± 0.5 **	33.3 ± 0.5 **
	(RIM)	33.0 ± 0.6	33.0 ± 0.7	33.2 ± 0.7	33.3 ± 0.7	33.3 ± 0.7 **	33.4 ± 0.7 **
	(CM)	33.2 ± 0.8	33.2 ± 0.7	33.2 ± 0.6	33.2 ± 0.7	33.2 ± 0.7	33.2 ± 0.7
Left Ankle	(ILM)	32.7 ± 1.5	32.7 ± 1.5	33.0 ± 1.3	33.1 ± 1.3 *	33.3 ± 1.3 **	33.4 ± 1.2 **
	(RIM)	32.7 ± 1.1	32.6 ± 1.1	32.9 ± 1.1	33.1 ± 1.0	33.2 ± 1.0 *	33.3 ± 1.0 **
	(CM)	33.4 ± 1.5	33.5 ± 1.5	33.6 ± 1.5	33.5 ± 1.4	33.5 ± 1.4	33.5 ± 1.4
Right Ankle	(LIM)	32.7 ± 1.6	32.8 ± 1.5	33.0 ± 1.4	33.2 ± 1.3	33.3 ± 1.2 *	33.5 ± 1.1 *
	(RIM)	32.8 ± 1.1	32.7 ± 1.2	33.0 ± 1.2	33.2 ± 1.1	33.3 ± 1.2 *	33.4 ± 1.0 **
	(CM)	33.3 ± 1.9	33.5 ± 1.9	33.5 ± 1.9	33.5 ± 1.8	33.5 ± 1.8	33.4 ± 1.7
Left Prox Ft	(LIM)	32.2 ± 2.4	32.3 ± 2.3	32.7 ± 2.2	32.8 ± 2.3	33.0 ± 2.3	33.1 ± 2.2 *
	(RIM)	32.1 ± 1.9	32.0 ± 1.8	32.4 ± 1.9	32.6 ± 1.9	32.9 ± 1.8	33.2 ± 1.5 **
	(CM)	33.6 ± 0.9	33.6 ± 0.9	33.7 ± 0.9	33.6 ± 0.9	33.6 ± 1.0	33.6 ± 1.0
Right Prox Ft	(LIM)	32.1 ± 2.3	32.2 ± 2.2	32.6 ± 2.2	32.8 ± 2.1	32.9 ± 2.1 *	33.1 ± 2.0 **
	(RIM)	32.1 ± 2.0	32.0 ± 2.0	32.3 ± 2.1	32.6 ± 2.1	32.8 ± 1.9	33.2 ± 1.5 **
	(CM)	33.6 ± 1.2	33.7 ± 1.3	33.7 ± 1.3	33.7 ± 1.2	33.7 ± 1.2	33.7 ± 1.2
Left Dist Ft	(LIM)	31.1 ± 3.7	31.3 ± 3.5	31.9 ± 3.6	32.2 ± 3.5	32.3 ± 3.5 *	32.7 ± 3.2 **
	(RIM)	30.8 ± 3.9	30.9 ± 3.5	31.6 ± 3.5	32.1 ± 3.2	32.6 ± 2.5	33.3 ± 1.8 **
	(CM)	32.9 ± 2.9	33.2 ± 2.8	33.2 ± 2.7	33.2 ± 2.6	33.2 ± 2.6	33.1 ± 2.5
Right Dist Ft	(LIM)	31.3 ± 3.4	31.5 ± 3.3	31.9 ± 3.4	32.2 ± 3.3 *	32.4 ± 3.1 **	32.9 ± 2.7 **
	(RIM)	30.8 ± 4.0	30.7 ± 3.8	31.4 ± 3.6	32 ± 3.2	32.7 ± 2.5	33.2 ± 1.9 **
	(CM)	32.8 ± 3.1	33.1 ± 3.1	33.2 ± 2.9	33.2 ± 2.9	33.2 ± 2.8	33.1 ± 2.8

Values presented are means (SD) °C Baseline (Pre), immediately after IM (Post 0), five minutes afterward (Post 5), ten minutes afterward (Post 10), 15 min afterward (Post 15), and 20 min afterward (Post 20). Low Lg = lower leg, Prox Ft = proximal foot, Dist Ft = distal foot, (L) = left side, (R) = right side Unit: °C * *p* < 0.05 vs. Pre. ** *p* < 0.01 vs. Pre.

**Table 2:** Skin temperature changes in the single stimulation of indirect moxibustion (IM) group (1× IM) and triple stimulation of IM group (3× IM).

Measurement Sites	Interventions	Pre	Post 0	Post 5	Post 10	Post 15	Post 20
Left Lg	(1× IM)	33.0 ± 0.7	33.0 ± 0.7	33.2 ± 0.7	33.3 ± 0.7 *	33.3 ± 0.7 **	33.4 ± 0.7 **
	(3× IM)	32.8 ± 0.6	33.0 ± 0.6	33.1 ± 0.6	33.1 ± 0.7 **	33.2 ± 0.7 **	33.3 ± 0.7 **
Right Lg	(1× IM)	33.0 ± 0.6	33.0 ± 0.7	33.2 ± 0.6	33.3 ± 0.7 **	33.4 ± 0.7 **	33.4 ± 0.7 **
	(3× IM)	32.9 ± 0.6	33.1 ± 0.6 *	33.2 ± 0.6 **	33.2 ± 0.7 **	33.3 ± 0.7 **	33.4 ± 0.7 **
Left Ankle	(1× IM)	32.8 ± 1.0	32.7 ± 1.0	33.0 ± 1.1	33.2 ± 1.0 *	33.3 ± 1.0 *	33.4 ± 1.0 **
	(3× IM)	32.4 ± .14	32.8 ± 1.2	33.0 ± 1.2 **	33.2 ± 1.3 **	33.4 ± 1.3 **	33.5 ± 1.3 **
Right Ankle	(1× IM)	32.9 ± 1.2	32.9 ± 1.2	33.1 ± 1.1	33.3 ± 1.1 *	33.4 ± 1.1 **	33.6 ± 1.0 **
	(3× IM)	32.7 ± 1.1	33.0 ± 1.0	33.2 ± 1.0 **	33.5 ± 1.2 **	33.6 ± 1.2 **	33.7 ± 1.2 **
Left Prox Ft	(1× IM)	32.0 ± 1.8	32.0 ± 1.8	32.3 ± 1.8	32.6 ± 1.8	32.8 ± 1.7 **	33.2 ± 1.4 **
	(3× IM)	31.9 ± 2.0	32.4 ± 2.0	32.8 ± 1.9 **	33.1 ± 2.1 **	33.2 ± 2.1 **	33.3 ± 2.1 **
Right Prox Ft	(1× IM)	32.1 ± 1.9	32.1 ± 1.9	32.3 ± 2.0	32.6 ± 1.9	32.9 ± 1.7 **	33.3 ± 1.4 **
	(3× IM)	32.2 ± 1.9	32.5 ± 1.9	32.9 ± 1.9 *	33.2 ± 2.0 **	33.4 ± 2.0 **	33.4 ± 2.0 **
Left Dist Ft	(1× IM)	30.3 ± 4.0	30.5 ± 3.7	31.1 ± 3.6	31.8 ± 3.2	32.4 ± 2.7 **	33.0 ± 2.2 **
	(3× IM)	31.0 ± 3.8	31.6 ± 3.4	32.3 ± 3.3	32.7 ± 3.4 **	32.9 ± 3.4 **	33.0 ± 3.3 **
Right Dist Ft	(1× IM)	30.5 ± 4.0	30.5 ± 3.8	31.1 ± 3.5	31.9 ± 3.1	32.6 ± 2.5 **	33.1 ± 2.0 **
	(3× IM)	31.3 ± 3.8	31.8 ± 3.4	32.5 ± 3.2	32.9 ± 3.2 **	33.1 ± 3.2 **	33.2 ± 3.0 **

Values presented are means (SD) °C Baseline (Pre), immediately after IM (Post 0), five minutes afterward (Post 5), ten minutes afterward (Post 10), 15 min afterward (Post 15), and 20 min afterward (Post 20). Low Lg = lower leg, Prox Ft = proximal foot, Dist Ft = distal foot, (L) = left side, (R) = right side. Unit: °C * *p* < 0.05 vs. Pre. ** *p* < 0.01 vs. Pre.

## References

[1] Mori H., Kuge H., Tanaka T.H., Taniwaki E., Ohsawa H. (2011). Is there a difference between the effects of single and triple indirect moxibustion stimulations on skin temperature changes of the posterior trunk surface?. Acupunct. Med..

[2] Mori H., Tanaka T.H., Kuge H., Sasaki K. (2012). Is there a difference between the effects of one-point and three-point indirect moxibustion stimulation on skin temperature changes of the posterior trunk surface?. Acupunct. Med..

[3] Chiba A., Nakanishi H., Chichibu S. (1997). Effect of indirect moxibustion on mouse skin. Am. J. Chin. Med..

[4] Sato A., Sato Y., Shimura M., Uchida S. (2000). Calcitonin gene-related peptide produces skeletal muscle vasodilation following antidromic stimulation of unmyelinated afferents in the dorsal root in rats. Neurosci. Lett..

[5] Kuge H., Mori H., Hatano Y. (2008). Influence of Fireless Moxibustion on QOL (SF-36^®^) in Elderly People. J. Jpn. Soc. Balneol. Climatol. Phys. Med..

[6] Su Y.S., Xin J.J., Yang Z.K., He W., Shi H., Wang X.Y., Hu L., Jing X.H., Zhu B. (2015). Effects of Different Local Moxibustion-Like Stimuli at Zusanli (ST36) and Zhongwan (CV12) on Gastric Motility and Its Underlying Receptor Mechanism. Evid. Based Complement. Altern. Med..

[7] Bao C., Zhang J., Liu J., Liu H., Wu L., Shi Y., Li J., Hu Z., Dong Y., Wang S. (2016). Moxibustion treatment for diarrhea-predominant irritable bowel syndrome: Study protocol for a randomized controlled trial. BMC Complement. Altern. Med..

[8] Jeon J.H., Cho C.K., Park S.J., Kang H.J., Kim K., Jung I.C., Kim Y.I., Lee S.H., Yoo H.S. (2017). A Feasibility Study of Moxibustion for Treating Anorexia and Improving Quality of Life in Patients With Metastatic Cancer: A Randomized Sham-Controlled Trial. Integr. Cancer Ther..

[9] Choi G.S., Han J.B., Park J.H., Oh S.D., Lee G.S., Bae H.S., Jung S.K., Cho Y.W., Ahn H.J., Min B.I. (2004). Effects of moxibustion to zusanli (ST36) on alteration of natural killer cell activity in rats. Am. J. Chin. Med..

[10] Su Y.S., Yang Z.K., Xin J.J., He W., Shi H., Wang X.Y., Hu L., Jing X.H., Zhu B. (2014). Somatosensory Nerve Fibers Mediated Generation of De-qi in Manual Acupuncture and Local Moxibustion-Like Stimuli-Modulated Gastric Motility in Rats. Evid. Based Complement. Altern. Med..

[11] Kuge H., Mori H., Tanaka T.H., Hanyu K., Morisawa T. (2013). Difference between the effects of one-site and three-site abdominal hot-stone stimulation on the skin-temperature changes of the lower limbs. J. Integr. Med..

[12] Kawakita K., Shinbara H., Imai K., Fukuda F., Yano T., Kuriyama K. (2006). How do acupuncture and moxibustion act?—Focusing on the progress in Japanese acupuncture research. J. Pharmacol. Sci..

[13] Chen M., Chen R., Xiong J., Chi Z., Sun J., Su T., Zhou M., Yi F., Zhang B. (2012). Evaluation of different moxibustion doses for lumbar disc herniation: Multicentre randomised controlled trial of heat-sensitive moxibustion therapy. Acupunct. Med..

[14] Chen R., Chen M., Xiong J., Chi Z., Zhang B., Tian N., Xu Z., Zhang T., Li W., Zhang W. (2013). Curative effect of heat-sensitive moxibustion on chronic persistent asthma: A multicenter randomized controlled trial. J. Tradit. Chin. Med..

[15] Chen R., Chen M., Xiong J., Chi Z., Zhou M., Su T., Sun J., Yi F., Zhang B. (2012). Is There Difference between the Effects of Two-Dose Stimulation for Knee Osteoarthritis in the Treatment of Heat-Sensitive Moxibustion?. Evid. Based Complement. Altern. Med..

[16] Xiao A., Chen R., Kang M., Tan S. (2012). Heat-sensitive moxibustion attenuates the inflammation after focal cerebral ischemia/reperfusion injury. Neural Regen. Res..

[17] Yamashita H., Tsukayama H., Tanno Y., Nishijo K. (1999). Adverse events in acupuncture and moxibustion treatment: A six-year survey at a national clinic in Japan. J. Altern. Complement. Med..

